# Lowered Resistance an Important Etiological Factor in Pneumonia

**Published:** 1918-11

**Authors:** 


### LOWERED RESISTANCE AN IMPORTANT ETIOLOGICAL FACTOR IN PNEUMONIA

Individuals in whom the resistance has been suddenly *lowered* are undoubtedly sometimes infected with pneumonia from the pneumococci that they themselves have harbored for long periods of time. The pneumococci, found in such individuals, that have not been exposed previously to cases of pneumonia, are said usually to belong to Class IV of the pneumococci, comprising organisms usually of low virulence; but if the resistance of the individual is just lowered by an attack of influenza, pneumonia is likely to result. Also, after exposure to cases of pneumonia or pneumonia convalescents, more virulent types of pneumococci are often found in the individuals so exposed, and if the natural resistance of such individuals becomes lowered from any cause, a virulent type of pneumonia is very likely to result.

Fatigue induced by overwork, and also by lack of sleep and worry, in connection with wet and cold, has been one reason for the excessive mortality from pneumonia in armies in the field. The lowered resistance from fatigue against pneumonia has been said to be due to the presence in the blood of katabolic products of muscular activity which have an injurious effect on the cells of the tissues in general, and on the leucocytes in particular, whose function may already have been disturbed by cold. It is well known that normal resistance to infection may be broken down in animals by fatigue, and phagocytosis lowered, and chemotactic power of the cells diminished. Excessive fatigue should, therefore, particularly at this time, be avoided as far as practicable in those commands suffering with respiratory disturbances.

The practical importance of fatigue as a predisposing cause of pneumonia was pointed out at the September meeting of the Research Society by Major Haven Emerson, who said that reports from France and the United States indicated that intensive, exhaustive drill was responsible for the increased susceptibility to respiratory infections amongst certain troops. Major Zinsser, who had investigated the epidemiology of pneumonia in a number of American camps, mentioned the " overwork factor " as being an important one. He said that there was a very distinct improvement in conditions when the drill was not exhaustive, and commended the method of the gradual introduction of the soldier to army life as a most excellent system in the prevention of respiratory infections. Regimental medical officers should lay these facts regarding the prevention of pneumonia before the regimental and company commanders in order to secure their co-operation.

